# Plasma Kynurenine to Tryptophan Ratio Is Negatively Associated with Linear Growth of Children Living in a Slum of Bangladesh: Results from a Community-Based Intervention Study

**DOI:** 10.4269/ajtmh.20-0049

**Published:** 2020-11-23

**Authors:** Md. Amran Gazi, Subhasish Das, Md. Abdullah Siddique, Md. Ashraful Alam, Shah Mohammad Fahim, Md. Mehedi Hasan, Farzana Hossaini, Md. Mamun Kabir, Zannatun Noor, Rashidul Haque, Mustafa Mahfuz, Tahmeed Ahmed

**Affiliations:** 1 Nutrition and Clinical Services Division, International Centre for Diarrheal Disease Research, Bangladesh (icddr,b), Dhaka, Bangladesh;; 2 Emerging Infection and Parasitology Laboratory, International Centre for Diarrheal Disease Research, Bangladesh (icddr,b), Dhaka, Bangladesh;; 3 James P. Grant School of Public Health, BRAC University, Dhaka, Bangladesh

## Abstract

Chronic exposure to infectious agents results in environmental enteric dysfunction—a significant contributor to childhood stunting. Low plasma tryptophan (TRP), increased kynurenine (KYN), and KYN–TRP (KT) ratio are associated with infections and chronic immune activation. We postulated that both these conditions are interlinked, and therefore aimed to identify the association between KT ratio and the linear growth of Bangladeshi children. A total of 480 stunted and at risk of being stunted children aged 12–18 months were enrolled and provided nutrition intervention for 90 days. Plasma samples were assessed using liquid chromatography tandem mass spectrometry to measure TRP and KYN concentrations. Multivariable linear regression with generalized estimating equations was applied to analyze association between the KT ratio and linear growth. Tryptophan, KYN, and KT ratio were significantly higher in stunted children than in children at risk of being stunted both at baseline and at the end of nutrition intervention. Following intervention, the median (interquartile range [IQR]) KYN concentration was significantly reduced from 4.6 (3.6, 5.4) µmol/L to 3.9 (0.3, 7.6) µmol/L, and median (IQR) KT ratio decreased from 104 (80.9, 131) to 92.8 (6.6, 247) in stunted children. We also found KT ratio to be negatively associated (coefficient = −0.7; 95% CI = −1.13, −0.26; *P*-value = 0.002) with linear growth. In addition, KYN and KT ratio were positively correlated with fecal neopterin and plasma C-reactive protein, whereas TRP was negatively correlated with both of these biomarkers and alpha-1-acid glycoprotein. Our findings imply that KT ratio is associated in the pathophysiology of stunting as well as with biomarkers of inflammation in Bangladeshi children.

## INTRODUCTION

Stunting is highly prevalent, especially in lower income countries, affecting an estimated 149 million children younger than 5 years, worldwide.^1^ It is an ailment where multiple pathological conditions manifested by linear growth faltering can increase morbidity, impaired brain development, as well as development of chronic diseases in adulthood.^2^ Short stature is common in low- and middle-income countries; thus, it often remains unrecognized and seems to be “normal” and taken for granted.^2^ It is difficult to identify visually and is not customarily evaluated by primary healthcare facilities. This exposes a noticeable number of children to the risk of being stunted.^2^ Living in poor hygiene and sanitary conditions may cause gut dysfunction, referred to as environmental enteric dysfunction (EED), a subclinical inflammatory condition of the small intestine.^3^ Growth retardation, gut microbiota alteration, and reduced vaccine responsiveness are thought to be the predominant consequences of EED.^4^ Exposures to environmental challenges impose metabolic stress that induces either nonspecific or more specific immune responses when infections occur.^5^ In the most severe infections, such exposures lead to the complete alteration of metabolic priorities, and to an underlying change in the requirements of protein, amino acids (AAs), and nitrogen.^5^

Tryptophan (TRP), a plant-derived essential AA (EAA), is needed to support growth and health in humans.^6^ It produces bioactive compounds via niacin and serotonin synthetic pathways, and serves as a precursor for protein synthesis.^6^ However, TRP is mostly catabolized by an enzyme called indoleamine 2,3-dioxygenase (IDO), induced by interferons, leading to the production of kynurenine (KYN).^7^ Reduction of TRP concentration and formation of the immunomodulatory metabolite KYN potentially impede T-cell proliferation and induce T-cell apoptosis.^7^ Immune activation thus gives rise to the formation of KYN and reduction of TRP, and a higher KYN–TRP (KT) ratio indicates a systemic immune response.^8^ The burden of infectious diseases is greater in children with stunting.^9^ Therefore, metabolic needs for these children require higher energy and AAs, which may divert EAAs to keep the immune system active at the expense of growth.^10^ For example, infestation with intestinal parasites increases the dietary requirements of lysine by 20% in undernourished children.^11^ Study also showed that chronic bacteria causing disease associated with wasting increased the protein turnover by nearly 40% when compared with uninfected subjects.^12^

Moreover, children in developing countries usually have limited access to animal source foods which are the richest sources of EAAs.^13^ Using a metabolomic approach, a study in rural Malawi showed that more than 80% children with stunting had reduced levels (15–20%) of all nine EAAs, compared with children with normal growth.^14^ Dietary restriction of these EAAs and their effects have also been explored in animal studies.^10^ Restriction of dietary TRP resulted in severe growth retardation of young rats and pregnant rats, thus deprived of dietary TRP-delivered growth-retarded newborns.^15^ Consequently, it was seen that mice with a mutation in TRP transport pathway suffered from malnutrition and that supplementation of TRP could mitigate associated problems.^16^

The KT ratio has been used as a systemic inflammatory marker in conditions such as sepsis, type 2 diabetes, obesity, inflammatory bowel disease, and immunodeficiency syndrome.^17–21^ The KT ratio has also been found to be positively associated with the growth deficits in children in Peru and Tanzania.^22^ In addition, serotonin/TRP and KT ratios were found to be positively correlated with gut permeability in Malawian children.^23^ Tryptophan, after being transformed to KYN or serotonin, remains no longer available to synthesize protein; hence, their ratios to TRP can be used as a proxy indicator of inflammatory conditions. In children living in slum where infections occur synchronously, the cascade of immune response to these infections is presumed to have an impact on child development and growth.^24^ Therefore, we hypothesized that KT pathway–mediated inflammation that causes insufficiency in EAAs may be a limiting factor for linear growth in children. The present study aimed to assess the role of KYN pathway of TRP metabolism in linear growth of Bangladeshi children.

## MATERIALS AND METHODS

### Study site, participants, and ethical consideration.

To perform this analysis, we have used data from the ongoing Bangladesh EED. This study is being conducted among slum-dwelling residents in Mirpur, Dhaka, Bangladesh. The study protocol has already been published elsewhere.^25^ The primary objective of this study is to validate noninvasive biomarkers of EED and investigate the relationship of those biomarkers with stunting. Understanding the mechanism and pathophysiology of EED is also an important objective of the study. Children aged between 12 and 18 months were recruited for an intervention period of 3 months. The intervention included 150 mL of whole milk, an egg, micronutrient powder, and also nutritional counseling for 90 feeding days. The cohort consists of stunted (length-for-age *Z*-scores [LAZ] < −2) and at risk of stunting (LAZ < −1 to −2) children. Data were taken from 480 children for this particular analysis. The Institutional Review Board of International Centre for Diarrhoeal Disease Research, Bangladesh approved the research protocol (protocol no PR-16007). Informed written consents were obtained from parents/caregivers of the participants before enrolment of their children to the study.

### Data collection, specimen collection, and storage.

Trained field staff measured the anthropometry following standard operating procedures. Weights and lengths of the participants were measured before and after the nutritional intervention along with mother’s height using standard scales (Seca GmbH & Co. KG., Hamburg, Germany). Measuring equipment was calibrated daily to maintain quality. In addition, uniformity of anthropometric measurement was also ensured by providing refresher training. Finally, intra-class correlation coefficient was estimated every 3 months. The WHO anthropometry calculator was used to calculate the indices of nutritional status, namely, weight-for-age *Z*-score (WAZ), weight-for-height *Z*-score (WHZ), and LAZ, both at baseline and after nutrition intervention. Stool and blood samples were collected at enrolment and at the end of nutrition intervention. To separate the plasma, blood samples were centrifuged at 4,000 rotations per minute (rpm) for 10 minutes. Aliquots of stool and plasma were immediately frozen at −80°C, pending analysis.

### Determination of TRP and KYN concentrations and KT ratio calculation.

Reference compounds for TRP, KYN, and TRP-d5 were procured from Sigma (St. Louis, MO). Kynurenine-d4 was purchased from TLC pharmaceuticals (Newmarket, Ontario, Canada). Acetonitrile, methanol, tetrahydrofuran, isopropanol, formic acid, ammonium formate, and other reagents or solvents were high-performance liquid chromatography grade or analytical grade. The mobile phase A contained acetonitrile:tetrahydrofuran:25 mM ammonium formate:formic acid in a ratio of 9:75:16:0.3 (v), whereas the mobile phase B contained acetonitrile:100 mM ammonium formate in a ratio of 20:80. Standards for L-TRP and L-KYN were made at 1 mg/mL in 50% (vol/vol) acetonitrile in water. Serial dilutions were prepared in water to reach a seven-point calibration curve with ranges of TRP (10–1,000 ng/mL) and KYN (2–200 ng/mL). The internal standard was prepared with TRP-d5 and KYN-d4 at a concentration of 250 ng/mL and 50 ng/mL, respectively.

20 μL human plasma samples were de-proteinized by adding methanol/acetonitrile (1:1) solvent. Then, 100 μL internal standard working solution was added, followed by vortexing for 10 minutes and centrifugation at 13,000 rpm for 10 minutes. The supernatant was separated and filtered with a 0.2-μm nylon filter. Finally, 10 μL samples were administered into the liquid chromatography with tandem mass spectrometry (LC-MS/MS) system. Flow rate for the system was set at 0.6 mL/minute, and each sample had run time of 18.5 minutes. A random standard solution was run after every 10 samples as a measure of quality control. Intrada AA 3 µm, 100 × 3.0 mm column (Chrom Tech, Apple Valley, MN) was used, and an LCMS-8050, a triple quadrupole LC-MS/MS (Shimadzu Corporation, Kyoto, Japan) was applied in this work. Overall, optimization process of KYN and TRP measurements was followed as described previously.^26,27^ The KT ratio was obtained by dividing plasma concentration of KYN (μmol/L) by the TRP concentration (μmol/L) and multiplying the quotient by 1,000.^17,21^ In this study, the KT ratio was used as a proxy of IDO activity.

### Analyses of plasma and stool biomarkers by ELISA.

Plasma samples were analyzed for C-reactive protein (CRP) (Immundiagnostik, Bensheim, Germany), alpha-1-acid glycoprotein (AGP) (Alpco, Salem, NH), and ferritin (ORGENTEC Diagnostika GmbH, 55129 Mainz, Germany) by using commercial ELISA kits. Zinc levels were determined by atomic absorption spectometry method. Stool samples were analyzed for neopterin (NEO) (GenWay, San Diego, CA), myeloperoxidase (Alpco), alpha-1-antitrypsin (AAT) (Biovendor, Chandler, NC), calprotectin (BÜHLMANN fCAL, Schönenbuch, Switzerland), and Reg1B (TechLab, Blacksburg, VA) using commercially available ELISAs according to kit manuals. The concentrations of each of the biomarkers were calculated against standards provided by the manufacturers.

### Statistical analyses.

Socioeconomic characteristics and demographic factors were described using frequencies with proportions when variables were categorical. Means and SDs were reported when quantitative variables were symmetrically distributed, whereas medians and interquartile ranges were used when quantitative variables were asymmetrically distributed. Differences in KYN, TRP, KT ratio, and other related variables were examined both pre- and post-intervention using Student’s *t*-test when variables were continuous and parametric, whereas the Mann–Whitney or Wilcoxon tests were used for nonparametric and continuous variables.

Correlations among the KYN, TRP, and KT ratio with the biomarkers of EED, inflammation, and micronutrient status were examined using Spearman’s correlation tests, both at baseline and endline. The correlation measures the strength and direction of association between different variables in our analysis. In this case, *P* < 0.05 was considered as a measure of significance. Multivariable linear regression with generalized estimating equations (GEEs) was applied to test the association between KT ratio and LAZ while adjusting for age, gender, water/sanitation, assets, maternal education, and income (WAMI) (a combined variable to measure household socioeconomic status, access to improved water/sanitation, assets, maternal education, and income), maternal height, micronutrients, and fecal and inflammatory biomarkers. In the GEE model, unstructured correlation matrix was used along with Gaussian family, and the link function was the identity. Correlation matrix was applied under independence model criterion (QIC) value and chosen based on the lowest quasi-likelihood. Variance inflation factor values were used to check the multicollinearity for all the models. Bivariate analysis was applied initially to identify the unadjusted effect of each explanatory variable on the outcome variable. Covariates were eventually selected for multivariable models if their association have significance of < 0.2 with the outcome. Multivariable analyses were then applied using GEEs to test the associations considering adjustment for potential confounders. A probability of < 0.05 was regarded statistically ignificant, and the strength of association was measured by the coefficient values and their 95% CIs.

The GEE method allows specification of a working correlation matrix for the within-subject correlation of repeated responses collected from the same participants over time that eventually produced unbiased and more efficient regression parameters. *R* version 3.5.1 was used for all the statistical analyses performed (https://www.r-project.org).

## RESULTS

### Sociodemographic and clinical characteristics.

A total of 480 participants comprising 240 stunted and 240 at risk of stunting children were included in this study. The two groups differed significantly in age and gender. The mean (±SD) age of stunted and at risk of stunting children were 14.8 ± 2.12 months and 14.3 ± 2.13 months, respectively (Table 1). Proportion of females was higher in children at risk of stunting (54.6%) than in stunted children (44.6%). The mean (±SD) LAZ was −2.79 ± 0.63 for stunted children and −1.56 ± 0.29 for at risk of stunting participants. Sociodemographic variables including length, weight, WAMI score, and maternal height were significantly lower in stunted than in at risk of being stunted children. The stunted children were also significantly more underweight (WAZ −2.15 versus −1.32) and wasted (WHZ −1.03 versus −0.80) than at risk of stunting participants. Besides, AAT level was found to be significantly different, whereas other stool and plasma biomarker levels did not differ significantly in the two groups.

**Table 1:** Baseline characteristics of the stunted and at risk of being stunted children at enrollment

Indicators	Stunted (*n* = 240)	At risk of stunting (*n* = 240)	*P*-value
Sociodemographic variables			
Mean age (SD) (months)	14.8 (2.12)	14.3 (2.13)	0.03
Female gender, *n* (%)	107 (44.6)	131 (54.6)	0.028
Mean weight-for-age *Z* score (SD)	−2.15 (0.772)	−1.32 (0.665)	< 0.001
Mean length-for-age *Z* score (SD)	−2.79 (0.628)	−1.56 (0.285)	< 0.001
Mean weight-for-height *Z* score (SD)	−1.03 (0.854)	−0.806 (0.862)	0.005
Mean weight (SD) (kg)	7.80 (0.843)	8.41 (0.781)	< 0.001
Mean length (SD) (cm)	70.8 (2.77)	73.4 (2.35)	< 0.001
Mean WAMI score (SD)	0.565 (0.123)	0.603 (0.137)	0.002
Mean maternal height (SD) (cm)	148 (5.45)	150 (4.83)	< 0.001
Fecal biomarkers of EED			
Median AAT [Q1, Q3] (mg/g)	0.230 (0.0600, 0.530)	0.340 (0.125, 0.565)	0.005
Median calprotectin [Q1, Q3] (µg/g)	544 (307, 1,010)	529 (283, 979)	0.902
Median MPO [Q1, Q3] (ng/mL)	2,310 (1,330, 4,550)	2,190 (1,340, 4,060)	0.516
Median NEO [Q1, Q3] (nmol/L)	2,330 (1,110, 3,430)	2,190 (1,220, 3,660)	0.572
Median Reg1B [Q1, Q3] (µg/mL)	62.7 (33.3, 87.5)	66.4 (33.1, 92.5)	0.152
Markers of systematic inflammation			
Median AGP [Q1, Q3] (mg/dL)	94.7 (72.0, 121)	91.5 (66.1, 124)	0.328
Median CRP [Q1, Q3] (mg/L)	1.32 (0.403, 3.87)	1.50 (0.509, 5.59)	0.155
Micronutrients			
Median ferritin [Q1, Q3] (ng/mL)	10.5 (5.24, 21.1)	12.1 (6.53, 22.9)	0.139
Median zinc [Q1, Q3] (mg/L)	0.790 (0.720, 0.875)	0.790 (0.710, 0.860)	0.845

AAT = alpha-1-anti-trypsin; AGP = alpha-1-acid glycoprotein; CRP = C-reactive protein; MPO = myeloperoxidase; NEO = neopterin; WAMI, measures of household socioeconomic status, including access to improved water/sanitation, assets, maternal education, and income (range 0–1). Normal ranges of CRP, AGP, ferritin, and zinc, and normal values for MPO, NEO, and AAT in nontropical settings are given in Fahim et al.^30^

### Levels of plasma TRP, KYN, and KT ratio in study participants.

**Figure 1.**
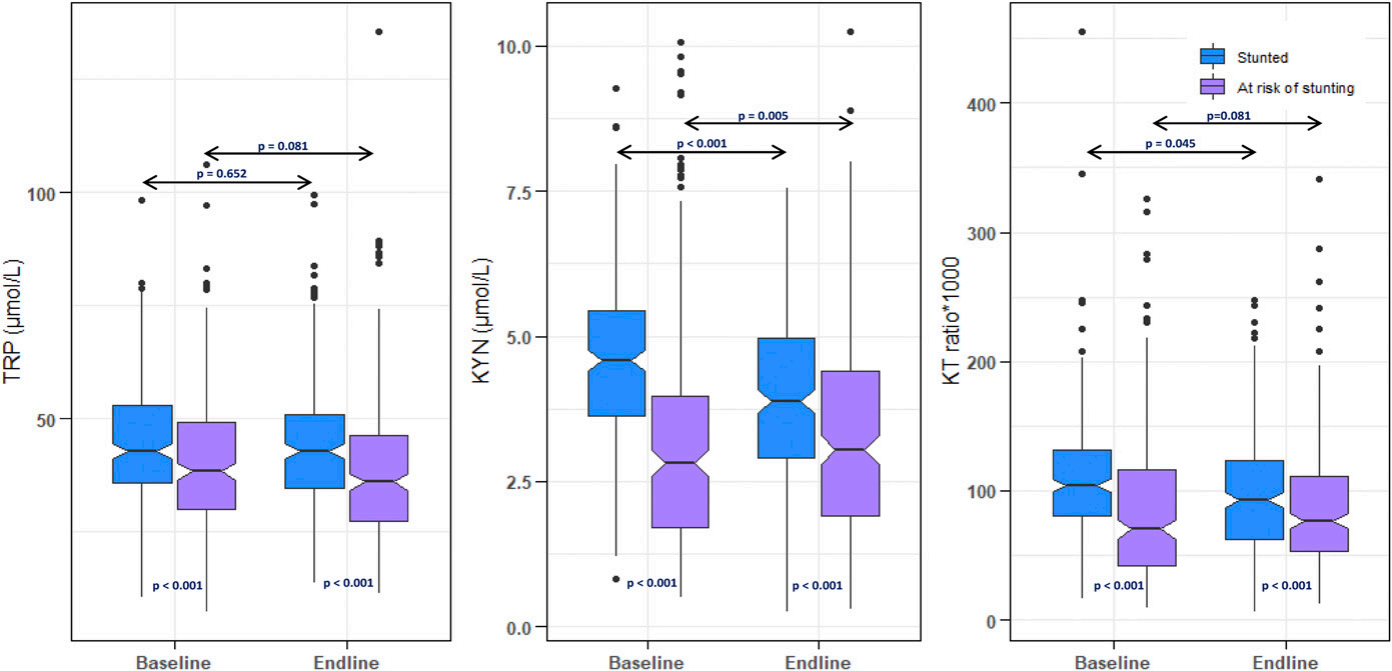
Baseline and endline concentrations of plasma tryptophan (TRP), kynurenine (KYN), and the KYN–TRP (KT) ratio in stunted and at risk of stunting children (or) the concentration of plasma TRP, KYN, and the KT ratio in stunted and at risk of stunting children, at baseline and endline. This figure appears in color at www.ajtmh.org.

Concentrations of TRP, KYN, and KT ratio as well as their comparisons in two groups are presented in Figure 1. Levels of TRP, KYN, and KT ratios were significantly higher in stunted participants than in at risk of being stunting participants, both at baseline and endline. In addition, the median concentration of KYN had significantly reduced to 3.88 (0.26, 7.56) µmol/L at endline from 4.58 (3.64, 5.45) µmol/L at baseline, and the KT ratio was also significantly reduced to 92.8 (6.66, 247) from 104 (80.9, 131) for stunted participants. However, KT ratios were significantly higher in endline for at risk of stunting participants than for their counterparts. Significant differences were not found between baseline and endline TRP levels, both for stunted and at risk of stunting participants.

### Correlation of TRP, KYN, and KT ratio with stool and plasma biomarkers.

In this analysis, correlations between different indicators at baseline and endline were assessed using Spearman’s rank correlation coefficient. Significant negative correlation was found between TRP and NEO both at baseline and endline (correlation coefficient, baseline = −0.12; endline = −0.12), whereas significant positive correlation was found for KYN at baseline (correlation coefficient = 0.17). The KT ratio was positively correlated with NEO (coefficient, baseline = 0.23; endline = 0.10). The KT ratio was negatively correlated with Reg1B at both time points, while being negatively correlated only at baseline with calprotectin and AAT. Among the plasma biomarkers, CRP was correlated negatively with TRP and positively with KT ratio, both at baseline and endline. Negative correlation was found for AGP and TRP at both time points (Supplemental Tables S1 and S2).

### Association of KT ratio and other variables with LAZ.

The analysis is longitudinal, with sample and anthropometric data collected from the same participants both in pre- and post-intervention. All the children were considered including stunted and at risk of stunting to analyze the association of biomarkers with LAZ. Table 2 shows the association of KT ratio and other indicators with LAZ using GEEs. The KT ratio was found to be negatively associated with LAZ (coefficient = −0.7; 95% CI = −1.13, −0.26; *P*-value = 0.002) on adjustment with the aforementioned confounders. Here, negative coefficient (−0.7) suggests that the mean decrease of LAZ by −0.7 unit for each one unit increase in the KT ratio. Significant positive association was observed in ferritin (coefficient = 1.62; 95% CI = 0.32, 2.92; *P*-value = 0.015), maternal height (coefficient = 0.03; 95% CI = 0.02, 0.04; *P*-value = < 0.001), and the WAMI score (coefficient = 0.79; 95% CI = 0.28, 1.3; *P*-value = 0.002) with LAZ of the children. None of the EED and systemic inflammatory markers were found to be associated with LAZ of the children.

**Table 2:** Association of KT ratio and other variables with length-for-age *Z*-score score using generalized estimating equation at baseline and endline (*n* = 480)

Indicators	Unadjusted β (95% CI)	*P*-value	Adjusted β (95% CI)	*P*-value
Age (months)	0.03 (0.02, 0.03)	< 0.001	0.02 (0.02, 0.03)	< 0.001
Female gender	0.17 (0.03, 0.31)	0.015	0.14 (0, 0.27)	0.044
KT ratio	−0.8 (−1.24, −0.36)	< 0.001	−0.7 (−1.13, −0.26)	0.002
AAT (mg/g)	0.02 (−0.01, 0.06)	0.190	0.02 (−0.01, 0.06)	0.172
Myeloperoxidase (mg/mL)	−3.99 (−8.12, 0.14)	0.059	−2.09 (−6.08, 1.9)	0.305
Neopterin (mmol/L)	0.16 (−4.14, 4.45)	0.944	–	–
Calprotectin (g/g)	0.76 (−29.16, 30.67)	0.960	–	–
Reg1B (mg/mL)	−0.14 (−0.47, 0.19)	0.403	–	–
AGP (g/dL)	−0.41 (−0.95, 0.13)	0.135	−0.03 (−0.57, 0.51)	0.900
CRP (g/L)	1.27 (−0.83, 3.37)	0.236	–	–
Ferritin (µg/mL)	1.24 (−0.08, 2.56)	0.066	1.62 (0.32, 2.92)	0.015
Zinc (mg/L)	−0.03 (−0.22, 0.16)	0.744	–	–
Maternal height (cm)	0.03 (0.02, 0.05)	< 0.001	0.03 (0.02, 0.04)	< 0.001
WAMI score	1.09 (0.58, 1.61)	< 0.001	0.79 (0.28, 1.3)	0.002

KYN–TRP = kynurenine–tryptophan.

## DISCUSSION

Linear growth failure in children is the most common manifestation of undernutrition worldwide.^28^ Impaired immune dysfunction resulting in recurrent infections may contribute to the high mortality rate associated with stunting, the causative pathways, which are still poorly understood.^4^ Moreover, unbalanced dietary intake and the mechanism by which it affects intestinal homeostasis are largely unrevealed.^16^ Identification of the mechanism and pathways associated with stunting are thus critical to distinguish the interrelated factors and to identify targets for intervention and mitigation. The TRP–IDO–KYN immunometabolic axis is a matter of great interest. In this study, we have quantified and analyzed the KYN pathway activation, also its association with linear growth of Bangladeshi children. Tryptophan metabolites have already been demonstrated to be promising indicators of TRP degradation induced by interferons.^29^ Our study results revealed that increased plasma KT ratio was found to be significantly and negatively associated with linear growth of the children in Bangladesh. Our findings also demonstrated that KYN and KT ratio were significantly elevated in stunted children compared with those among the at risk of being stunted children. In addition, KYN concentrations and KT ratios declined in stunted children receiving nutritional supplementation. However, we did not have a control group of stunted children to prove whether this supplementation led to the decrease in KYN and KT ratio. Hence, a control group (who did not receive supplementation) and comparison is essential to prove the supplementation effect. Our study also revealed that level of AAT was found to be significantly higher in non-stunted than in stunted children, whereas other stool and plasma biomarker levels did not differ between the groups. Alpha-1-anti-trypsin is an acute phase protein that indicates increased intestinal permeability and protein loss.^30^ Increased AAT in non-stunted children might explain the lower level of TRP found in this group. However, this finding complicates the overall notion of a physiologic pathway between intestinal permeability and chronic systemic inflammation.

Elevated KYN and KT ratio are markers of increased IDO activity.^31^ Increased KT ratio has shown to be associated with stunting earlier in other settings.^22,32^ Moreover, TRP and KYN/TRP ratio associated with linear growth failure are accordant with prior studies that showed growth outcomes to be linked with several determinants including inadequate intake of nutrition,^33,34^ EED,^35,36^ intestinal permeability, and disruption of the gut microbiota.^37^ Tryptophan metabolism mainly occurs via KYN pathway in the liver where the rate-limiting and first step is catalyzed by the hepatic TRP 2,3-dioxygenase (TDO) enzyme or the ubiquitous IDO.^38^ Microbial TRP uptake and metabolism may subvert this pathway.^39^ Whereas TDO is inherently active, IDO is activated by inflammatory cytokines. Therefore, in conditions with increased inflammation, TRP metabolism is switched from hepatic to extrahepatic tissues.^38^ Participants in the current study were slum dwellers and lived in a poor socioeconomic environment where frequent infection with pathogen is the norm. One of the adverse effects of elevated degradation and depletion of TRP in the body is the suppression of T-cell proliferation which is particularly evident in HIV infection.^40^ Other manifestations include weight loss, cognitive impairment, and mood disturbances. Cachexia or weight loss has also been found to be associated with low TRP levels in neoplasia.^41^ Increased catabolism of TRP through the KYN pathway may shift this EAA away from protein synthesis, and thus have contribution in muscle wasting and weight loss.^41^ Partitioning of AAs occur during immune system activation because of the competition between protein gain and utilization by the system.^42^ When supply of EAAs such as methionine, cysteine, threonine, and TRP is limited, there is greater competition associated with the immune system for the AAs.^42^ The condition may also be associated with low socioeconomic conditions, intestinal infections, and multiple dietary factors that cause changes in microbiome. A murine model study found that T cells cultured with IDO producing cells or low TRP media have lowered cell growth and increased cell apoptosis.^17^

Limitation of TRP in the diet could possibly have adverse effects on child growth through reduction of protein synthesis.^23^ Nutritional deficiencies and other sociodemographic variables can also weaken the immune system, with potential effects on IDO activity.^43^ Children who are stunted often have insufficient TRP in their diets, and it has been exhibited that stunted children have lower levels of all nine EAAs in the serum, including TRP.^14^ Low serum TRP was found associated with biomarkers of gut barrier disruption and systemic inflammation and a predictor of growth failure in boys.^44^ Consequently, the syntheses of both proteins and lipids are restricted because of the mammalian target of rapamycin complex 1 (mTORC1) gene being repressed. mTORC1 is a master growth regulator, and the availability of AAs is exquisitely sensed via this pathway, for synthesis of proteins.^10^ Tryptophan activates mTORC1 and enhances the expression of tight junction proteins, involved in maintaining intestinal barrier function.^6^ Stunted children in lower income countries have scarce intake of EAAs, and this might hinder growth through the mTORC1 pathway.

In the current study, LAZ was found to be positively associated with plasma ferritin, maternal height, and WAMI score. Ferritin indicates the measure of iron stores in the body, if there is no concurrent infection. We adjusted the concentrations using AGP and CRP before analyses. As iron stores diminish, the ferritin value will decrease and cause immune disruption and stunting. Moreover, previous findings showed that intestinal ferritin loss may occur because of disruption of gut barrier function.^30^ Study also reported negative relationship between intestinal permeability and serum ferritin concentrations in young children and infants.^45^ The results of the WAMI score, maternal height, and stunting in this study are also consistent with previously published literature.^46,47^ None of the EED and systematic inflammatory biomarkers were associated with stunting. These results of EED and inflammatory biomarkers with linear growth are in line with other studies in same settings.^48,49^

We also assessed the correlation of TRP, KYN, and KT ratio with biomarkers of EED, systematic inflammatory biomarkers, and micronutrients. Our study revealed the positive correlation of NEO with KT ratio, both in baseline and endline, and KYN in baseline. Negative correlation was found for NEO with TRP at both time points. In some studies, concentrations of NEO were specifically quantified in different diseased conditions and showed a positive correlation with the KT ratio and KYN, and inversely with TRP.^7,21,50,51^ Neopterin is a marker of gut inflammation and immune system activation that is released by macrophages or monocytes, which activates the IDO pathway upon stimulation by interferon-gamma.^52^ So a rise in the NEO level gives a good estimate of the endogenous production of interferon-gamma and thus upregulation of the KYN pathway in EED and undernutrition. Possible mechanisms by which EED could influence TRP metabolism is through increased TRP breakdown by altered gut microbiota or through alterations in TRP transport by AA transporters. It is noted that dietary TRP is mostly absorbed by epithelial enterocytes using the neutral amino acid transporter/angiotensin-converting enzyme 2 transport pathway.^16^ One murine model study reported that deficiency in ACE2 results in increased risk of intestinal inflammation. Restoring TRP levels reduced inflammation and increased the production of antimicrobial peptides, indicating that ACE2 regulates dietary AA homeostasis, inflammation, gut microbial ecology, and innate immunity in a TRP-dependent manner.^16^ Our study result suggests that manipulation of TRP signaling may be pertinent to the pathophysiology of EED.^22,53^ A possible role of TRP signaling in regulating the villus structure and nutrient-sensing capability of immune cell has also been observed.^54^ A negative correlation was found for Reg1B with KYN and KT ratio both at baseline and endline, and again, negative correlation was found for AAT and calprotectin with KYN and KT ratio only at the endline. These negative correlations may be explained by the transient nature of EED markers in young children and need further elucidation with the KYN pathway. Moreover, KT ratio was positively correlated with AGP and CRP—biomarkers and measures of systemic inflammation. This finding is compatible with current thinking that intestinal infection, enterocyte function, and systemic immune response are closely related to EED.^22^ A recent study has shown that patients with flaky paint dermatosis had lower plasma TRP, and the authors had explained that the activity of IDO may be enhanced, because of increased CRP levels.^55^

## STRENGTHS AND LIMITATIONS

There were some limitations in this study. First, IDO expression was not measured directly in the present study. However, studies have manifested that IDO activity is estimated best by the KT ratio. Second, increased expressions of interferon-gamma, interleukin-6, and interleukin-10 have been shown to be associated with increased catabolism of TRP by IDO. However, none of the mentioned cytokines were measured in our study. Besides this, the target population was children who were stunted or were at risk of stunting, and therefore by definition were not completely healthy. Finally, inability to include some of the important variables, such as birth weight, breastfeeding status, and morbidity were also major limitations of this study. The strength of this study includes a larger sample size. In addition, all the biomarkers were tested in a single batch to reduce potential batch effects and inter-tester variation.

In conclusion, the KT ratio was found to be significantly associated with growth in Bangladeshi children. In this study, as an estimation of increased IDO activity, the KT ratio came out as a possible measurement of childhood stunting. However, important questions remain to be unraveled that how TRP and KYN or KT ratio is reduced or elevated in the context of undernutrition and could be explored further in prospective studies. This alteration in the TRP pathway may reflect the effect of environmental stressors such as chronic inflammation, EED, low protein intake, or variations in the intestinal microbiota, because all of these can significantly affect the growth.

## Supplemental tables

Supplemental materials

## References

[1] UNICEF, 2018 Malnutrition Rates Remain Alarming: Stunting Is Declining Too Slowly while Wasting Still Impacts the Lives of Far Too Many Young Children. New York, NY: UNICEF.

[2] De Onis M Branca F, 2016 Childhood stunting: a global perspective. Matern Child Nutr 12: 12–26. 27187907 10.1111/mcn.12231 PMC5084763

[3] Mbuya MN Humphrey JH, 2016 Preventing environmental enteric dysfunction through improved water, sanitation and hygiene: an opportunity for stunting reduction in developing countries. Matern Child Nutr 12: 106–120. 10.1111/mcn.12220 PMC5019251 26542185

[4] Bourke CD Berkley JA Prendergast AJ, 2016 Immune dysfunction as a cause and consequence of malnutrition. Trends Immunol 37: 386–398. 27237815 10.1016/j.it.2016.04.003 PMC4889773

[5] WHO, United Nations University, 2007 Protein and Amino Acid Requirements in Human Nutrition. Geneva, Switzerland: World Health Organization. 18330140

[6] Wang H Ji Y Wu G Sun K Sun Y Li W Wang B He B Zhang Q Dai Z, 2015 L-tryptophan activates mammalian target of rapamycin and enhances expression of tight junction proteins in intestinal porcine epithelial cells. J Nutr 145: 1156–1162. 25878205 10.3945/jn.114.209817

[7] Suzuki Y Suda T Furuhashi K Suzuki M Fujie M Hahimoto D Nakamura Y Inui N Nakamura H Chida K, 2010 Increased serum kynurenine/tryptophan ratio correlates with disease progression in lung cancer. Lung Cancer 67: 361–365. 19487045 10.1016/j.lungcan.2009.05.001

[8] Harper KM Mutasa M Prendergast AJ Humphrey J Manges AR, 2018 Environmental enteric dysfunction pathways and child stunting: a systematic review. PLoS Negl Trop Dis 12: e0006205. 29351288 10.1371/journal.pntd.0006205 PMC5792022

[9] Walson JL Berkley JA, 2018 The impact of malnutrition on childhood infections. Curr Opin Infect Dis 31: 231–236. 29570495 10.1097/QCO.0000000000000448 PMC6037284

[10] Semba RD Trehan I Gonzalez-Freire M Kraemer K Moaddel R Ordiz MI Ferrucci L Manary MJ, 2016 Perspective: the potential role of essential amino acids and the mechanistic target of rapamycin complex 1 (mTORC1) pathway in the pathogenesis of child stunting. Adv Nutr 7: 853–865. 27633102 10.3945/an.116.013276 PMC5015042

[11] Pillai RR Elango R Ball RO Kurpad AV Pencharz PB, 2015 Lysine requirements of moderately undernourished school-aged Indian children are reduced by treatment for intestinal parasites as measured by the indicator amino acid oxidation technique. J Nutr 145: 954–959. 25761501 10.3945/jn.114.208439

[12] Paton NI Angus B Chaowagul W Simpson AJ Suputtamongkol Y Elia M Calder G Milne E White NJ Griffin GE, 2001 Protein and energy metabolism in chronic bacterial infection: studies in melioidosis. Clin Sci 100: 101–110. 11115424

[13] Dror DK Allen LH, 2011 The importance of milk and other animal-source foods for children in low-income countries. Food Nutr Bull 32: 227–243. 22073797 10.1177/156482651103200307

[14] Semba RD Shardell M Ashour FAS Moaddel R Trehan I Maleta KM Ordiz MI Kraemer K Khadeer MA Ferrucci L, 2016 Child stunting is associated with low circulating essential amino acids. EBioMedicine 6: 246–252. 27211567 10.1016/j.ebiom.2016.02.030 PMC4856740

[15] Sanfilippo S Imbesi R Sanfilippo JS, 1995 Effects of a tryptophan deficient diet on the morphology of skeletal muscle fibers of the rat. Preliminary observations atneuroendocrinological and submicroscopical levels. Ital J Anat Embryol 100: 131–141. 11322286

[16] Hashimoto T Perlot T Rehman A Trichereau J Ishiguro H Paolino M Sigl V Hanada T Hanada R Lipinski S, 2012 ACE2 links amino acid malnutrition to microbial ecology and intestinal inflammation. Nature 487: 477–481. 22837003 10.1038/nature11228 PMC7095315

[17] Darcy CJ Davis JS Woodberry T McNeil YR Stephens DP Yeo TW Anstey NM, 2011 An observational cohort study of the kynurenine to tryptophan ratio in sepsis: association with impaired immune and microvascular function. PLoS One 6: e21185. 21731667 10.1371/journal.pone.0021185 PMC3120841

[18] Rebnord EW Strand E Midttun Ø Svingen GF Christensen MH Ueland PM Mellgren G Njølstad PR Tell GS Nygård OK, 2017 The kynurenine: tryptophan ratio as a predictor of incident type 2 diabetes mellitus in individuals with coronary artery disease. Diabetologia 60: 1712–1721. 28612106 10.1007/s00125-017-4329-9 PMC5552838

[19] Favennec M Hennart B Caiazzo R Leloire A Yengo L Verbanck M Arredouani A Marre M Pigeyre M Bessede A, 2015 The kynurenine pathway is activated in human obesity and shifted toward kynurenine monooxygenase activation. Obesity 23: 2066–2074. 26347385 10.1002/oby.21199

[20] Ciorba MA, 2013 Indoleamine 2, 3 dioxygenase (IDO) in intestinal disease. Curr Opin Gastroenterol 29: 146–152. 23283180 10.1097/MOG.0b013e32835c9cb3 PMC3686557

[21] Huengsberg M Winer JB Gompels M Round R Ross J Shahmanesh M, 1998 Serum kynurenine-to-tryptophan ratio increases with progressive disease in HIV-infected patients. Clin Chem 44: 858–862. 9554499

[22] Kosek MN Mduma E Kosek PS Lee GO Svensen E Pan WK Olortegui MP Bream JH Patil C Asayag CR, 2016 Plasma tryptophan and the kynurenine–tryptophan ratio are associated with the acquisition of statural growth deficits and oral vaccine underperformance in populations with environmental enteropathy. Am J Trop Med Hyg 95: 928–937. 27503512 10.4269/ajtmh.16-0037 PMC5062803

[23] Semba RD Shardell M Trehan I Moaddel R Maleta KM Ordiz MI Kraemer K Khadeer M Ferrucci L Manary MJ, 2016 Metabolic alterations in children with environmental enteric dysfunction. Sci Rep 6: 28009. 27294788 10.1038/srep28009 PMC4904796

[24] Platts-Mills JA Babji S Bodhidatta L Gratz J Haque R Havt A McCormick BJ McGrath M Olortegui MP Samie A, 2015 Pathogen-specific burdens of community diarrhoea in developing countries: a multisite birth cohort study (MAL-ED). Lancet Glob Health 3: e564–e575. 26202075 10.1016/S2214-109X(15)00151-5 PMC7328884

[25] Mahfuz M Das S Mazumder RN Rahman MM Haque R Bhuiyan MMR Akhter H Sarker MSA Mondal D Muaz SSA, 2017 Bangladesh Environmental Enteric Dysfunction (BEED) study: protocol for a community-based intervention study to validate non-invasive biomarkers of environmental enteric dysfunction. BMJ Open 7: e017768. 10.1136/bmjopen-2017-017768 PMC5724211 28801442

[26] Möller M Du Preez JL Harvey BH, 2012 Development and validation of a single analytical method for the determination of tryptophan, and its kynurenine metabolites in rat plasma. J Chromatogr B 898: 121–129. 10.1016/j.jchromb.2012.04.030 22608808

[27] Sun Z Xing J Khoo PY Zhan Z, 2016 A Combined MRM and SIM Method for Direct Quantitative Determination of Amino Acids in Various Samples on LC/MS/MS. Sante Fe, NM: American Society for Mass Spectrometry.

[28] Prendergast AJ Humphrey JH, 2014 The stunting syndrome in developing countries. Paediatr Int Child Health 34: 250–265. 25310000 10.1179/2046905514Y.0000000158 PMC4232245

[29] Werner ER Bitterlich G Fuchs D Hausen A Reibnegger G Szabo G Dierich MP Wachter H, 1987 Human macrophages degrade tryptophan upon induction by interferon-gamma. Life Sci 41: 273–280. 3110526 10.1016/0024-3205(87)90149-4

[30] Fahim SM Das S Sanin KI Gazi MA Mahfuz M Islam MM Ahmed T, 2018 Association of fecal markers of environmental enteric dysfunction with zinc and iron status among children at first two years of life in Bangladesh. Am J Trop Med Hyg 99: 489–494. 29893201 10.4269/ajtmh.17-0985 PMC6090336

[31] Pett SL Kunisaki KM Wentworth D Griffin TJ Kalomenidis I Nahra R Montejano Sanchez R Hodgson SW Ruxrungtham K Dwyer D, 2017 Increased indoleamine-2, 3-dioxygenase activity is associated with poor clinical outcome in adults hospitalized with influenza in the INSIGHT FLU003Plus study. Open Forum Infect Dis 5: ofx228. 29322062 10.1093/ofid/ofx228 PMC5753217

[32] Moreau GB Ramakrishnan G Cook HL Fox TE Nayak U Ma JZ Colgate ER Kirkpatrick BD Haque R Petri WA Jr., 2019 Childhood growth and neurocognition are associated with distinct sets of metabolites. EBioMedicine 44: 597–606. 31133540 10.1016/j.ebiom.2019.05.043 PMC6604877

[33] Rah JH Akhter N Semba RD De Pee S Bloem MW Campbell A Moench-Pfanner R Sun K Badham J Kraemer K, 2010 Low dietary diversity is a predictor of child stunting in rural Bangladesh. Eur J Clin Nutr 64: 1393–1398. 20842167 10.1038/ejcn.2010.171

[34] Ghosh S Suri D Uauy R, 2012 Assessment of protein adequacy in developing countries: quality matters. Br J Nutr 108: S77–S87. 23107551 10.1017/S0007114512002577

[35] Crane RJ Jones KD Berkley JA, 2015 Environmental enteric dysfunction: an overview. Food Nutr Bull 36: S76–S87. 25902619 10.1177/15648265150361S113 PMC4472379

[36] Merchant A Jones C Kiure A Kupka R Fitzmaurice G Herrera M Fawzi W, 2003 Water and sanitation associated with improved child growth. Eur J Clin Nutr 57: 1562–1568. 14647221 10.1038/sj.ejcn.1601725

[37] Charbonneau MR O’Donnell D Blanton LV Totten SM Davis JC Barratt MJ Cheng J Guruge J Talcott M Bain JR, 2016 Sialylated milk oligosaccharides promote microbiota-dependent growth in models of infant undernutrition. Cell 164: 859–871. 26898329 10.1016/j.cell.2016.01.024 PMC4793393

[38] Badawy AA, 2017 Kynurenine pathway of tryptophan metabolism: regulatory and functional aspects. Int J Tryptophan Res 10: 1178646917691938. 28469468 10.1177/1178646917691938 PMC5398323

[39] Ahmed T Auble D Berkley JA Black R Ahern PP Hossain M Hsieh A Ireen S Arabi M Gordon JI, 2014 An evolving perspective about the origins of childhood undernutrition and nutritional interventions that includes the gut microbiome. Ann New York Acad Sci 1332: 22–38. 25118072 10.1111/nyas.12487 PMC4514967

[40] Vujkovic-Cvijin I Dunham RM Iwai S Maher MC Albright RG Broadhurst MJ Hernandez RD Lederman MM Huang Y Somsouk M, 2013 Dysbiosis of the gut microbiota is associated with HIV disease progression and tryptophan catabolism. Sci Transl Med 5: 193ra91. 10.1126/scitranslmed.3006438 PMC4094294 23843452

[41] Chen Y Guillemin GJ, 2009 Kynurenine pathway metabolites in humans: disease and healthy states. Int J Tryptophan Res 2: 1–19. 22084578 10.4137/ijtr.s2097 PMC3195227

[42] Kampman-van de Hoek E Jansman AJ van den Borne JJ van der Peet-Schwering CM van Beers-Schreurs H Gerrits WJ, 2015 Dietary amino acid deficiency reduces the utilization of amino acids for growth in growing pigs after a period of poor health. J Nutr 146: 51–58. 26609170 10.3945/jn.115.216044

[43] Badawy AA, 2017 Tryptophan availability for kynurenine pathway metabolism across the life span: control mechanisms and focus on aging, exercise, diet and nutritional supplements. Neuropharmacology 112: 248–263. 26617070 10.1016/j.neuropharm.2015.11.015

[44] Guerrant RL Leite AM Pinkerton R Medeiros PH Cavalcante PA DeBoer M Kosek M Duggan C Gewirtz A Kagan JC, 2016 Biomarkers of environmental enteropathy, inflammation, stunting, and impaired growth in children in northeast Brazil. PLoS One 11: e0158772. 27690129 10.1371/journal.pone.0158772 PMC5045163

[45] Berant M Khourie M Menzies IS, 1992 Effect of iron deficiency on small intestinal permeability in infants and young children. J Pediatr Gastroenterol Nutr 14: 17–20. 1573506 10.1097/00005176-199201000-00004

[46] Psaki SR Seidman JC Miller M Gottlieb M Bhutta ZA Ahmed T Ahmed AS Bessong P John SM Kang G, 2014 Measuring socioeconomic status in multicountry studies: results from the eight-country MAL-ED study. Popul Health Metr 12: 8. 24656134 10.1186/1478-7954-12-8 PMC4234146

[47] MAL-ED Network Investigators, 2017 Childhood stunting in relation to the pre-and postnatal environment during the first 2 years of life: the MAL-ED longitudinal birth cohort study. PLoS Med 14: e1002408. 29069076 10.1371/journal.pmed.1002408 PMC5656304

[48] Campbell RK Schulze K Shaikh S Mehra S Ali H Wu L Raqib R Baker S Labrique A West KP Jr., 2017 Biomarkers of environmental enteric dysfunction among children in rural Bangladesh. J Pediatr Gastroenterol Nutr 65: 40–46. 28644348 10.1097/MPG.0000000000001557 PMC5492885

[49] Hossain M Nahar B Haque M Mondal D Mahfuz M Naila NN Gazi M Hasan M Haque NMS Haque R, 2019 Serum adipokines, growth factors, and cytokines are independently associated with stunting in Bangladeshi children. Nutrients 11: 1827. 10.3390/nu11081827 PMC6723106 31394828

[50] Dusek T Orhalmi J Sotona O Krcmova LK Javorska L Dolejs J Paral J, 2018 Neopterin, kynurenine and tryptophan as new biomarkers for early detection of rectal anastomotic leakage. Videosur Other Miniinvasive Tech 13: 44–52. 10.5114/wiitm.2018.73363 PMC5890852 29643957

[51] Murr C Gerlach D Widner B Dierich MP Fuchs D, 2001 Neopterin production and tryptophan degradation in humans infected by *Streptococcus pyogenes*. Med Microbiol Immunol 189: 161–163. 11388614 10.1007/s430-001-8023-3

[52] Zuo H Ueland PM Ulvik A Eussen SJ Vollset SE Nygård O Midttun Ø Theofylaktopoulou D Meyer K Tell GS, 2016 Plasma biomarkers of inflammation, the kynurenine pathway, and risks of all-cause, cancer, and cardiovascular disease mortality: the Hordaland health study. Am J Epidemiol 183: 249–258. 26823439 10.1093/aje/kwv242 PMC4753283

[53] Metz R Rust S DuHadaway JB Mautino MR Munn DH Vahanian NN Link CJ Prendergast GC, 2012 IDO inhibits a tryptophan sufficiency signal that stimulates mTOR: a novel IDO effector pathway targeted by D-1-methyl-tryptophan. Oncoimmunology 1: 1460–1468. 23264892 10.4161/onci.21716 PMC3525601

[54] Louis-Auguste J Besa E Zyambo K Munkombwe D Banda R Banda T Watson A Mayneris-Perxachs J Swann J Kelly P, 2019 Tryptophan, glutamine, leucine, and micronutrient supplementation improves environmental enteropathy in Zambian adults: a randomized controlled trial. Am J Clin Nutr 110: 1240–1252. 31504110 10.1093/ajcn/nqz189 PMC6821547

[55] Maltos AL Portari GV Moraes GV Monteiro MCR Vannucchi H da Cunha DF, 2015 Niacin metabolism and indoleamine 2, 3-dioxygenase activation in malnourished patients with flaky paint dermatosis. Nutrition 31: 890–892. 25933499 10.1016/j.nut.2014.12.023

