# Supplementary material for: Plasma Kynurenine to Tryptophan Ratio Is Negatively Associated with Linear Growth of Children Living in a Slum of Bangladesh: Results from a Community-Based Intervention Study
Source: Am J Trop Med Hyg. 2020 Nov 23;104(2):766–73. doi: 10.4269/ajtmh.20-0049 (PMC7866305; doi:10.4269/ajtmh.20-0049)
Supplement: Supplementary file 1 [file tpmd200049.SD1.pdf]

Supplemental Table S1: Spearman rank correlation between different indicators at baseline

	TRP	KYN	KT ratio	AAT	Calprotectin	MPO	NEO	Reg1B	AGP	CRP	Ferritin	zinc
TRP	1											
KYN	0.276*	1										
KT ratio	-0.354*	0.755*	1									
AAT	0.034	-0.114*	-0.133*	1								
Calprotectin	-0.058	-0.150*	-0.126*	0.274*	1							
MPO	0.009	-0.014	-0.028	0.353*	0.371*	1						
NEO	-0.115*	0.165*	0.225*	0.214*	0.048	0.019	1					
Reg1B	0.149*	-0.057	-0.164*	0.042	0.091*	0.266*	-0.050	1				
AGP	-0.162*	-0.014	0.055	0.036	0.147*	0.159*	-0.016	0.005	1			
CRP	-0.226*	0.005	0.169*	0.039	0.099*	0.168*	0.018	-0.003	0.431*	1		
Ferritin	-0.117*	0.036	0.115*	-0.084	-0.111*	-0.088	0.044	-0.081	0.115*	0.181*	1	
zinc	0.122*	-0.013	-0.095*	0.071	0.036	0.063	-0.004	0.046	-0.091*	-0.182*	-0.060	1

* Sign shows the statistical significance $P<0.05$

Supplemental Table S2: Spearman rank correlation between different indicators at endline

	TRP	KYN	KT ratio	AAT	Calprotectin	MPO	NEO	Reg1B	AGP	CRP	Ferritin	zinc
TRP	1											
KYN	0.397*	1										
KT ratio	-0.259*	0.726*	1									
AAT	0.076	0.042	-0.032	1								
Calprotectin	-0.003	0.024	0.028	0.266*	1							
MPO	-0.020	-0.041	-0.033	0.364*	0.499*	1						
NEO	-0.158*	-0.003	0.103*	-0.001	-0.055	0.052	1					
Reg1B	-0.035	-0.133*	-0.109*	0.071	0.116*	0.185*	0.173*	1				
AGP	-0.182*	-0.033	0.073	0.081	0.116*	0.066	0.020	-0.006	1			
CRP	-0.214*	-0.013	0.135*	0.038	0.149*	0.093*	-0.063	0.009	0.467*	1		
Ferritin	0.021	-0.016	-0.018	-0.154*	-0.099*	-0.132*	-0.074	0.07	0.097*	0.147*	1	
zinc	0.056	-0.020	-0.077	0.008	0.061	0.011	0.013	0.008	-0.119*	-0.207*	-0.096*	1

* Sign shows the statistical significance $P<0.05$
